# Analysis of saponins detoxification genes in *Ilyonectria mors-panacis* G3B inducing root rot of *Panax notoginseng* by RNA-Seq

**DOI:** 10.1007/s00203-021-02502-4

**Published:** 2021-08-04

**Authors:** Guohong Zeng, Jin Li, Yuxiu Ma, Qian Pu, Tian Xiao, Ruihuan Yang, Xiufang Hu

**Affiliations:** Zhejiang Province Key Laboratory of Plant Secondary Metabolism and Regulation, College of Life Science and Medicine, Zhejiang Sci-Tech University, Road 2, Xiasha, Hangzhou, 310018 China

**Keywords:** *Ilyonectria mors-panacis* G3B, *Panax notoginseng*, RNA-Seq, Root rot, Saponins detoxification genes

## Abstract

Saponins are kinds of antifungal compounds produced by *Panax notoginseng* to resist invasion by pathogens. *Ilyonectria mors-panacis* G3B was the dominant pathogen inducing root rot of *P. notoginseng*, and the abilities to detoxify saponins were the key to infect *P. notoginseng* successfully. To research the molecular mechanisms of detoxifying saponins in *I. mors-panacis* G3B, we used high-throughput RNA-Seq to identify 557 and 1519 differential expression genes (DEGs) in *I. mors-panacis* G3B with saponins treatments for 4H (Hours) and 12H (Hours) compared with no saponins treatments, respectively. Among these DEGs, we found 93 genes which were simultaneously highly expressed in *I. mors-panacis* G3B with saponins treatments for 4H and 12H, they mainly belong to genes encoding transporters, glycoside hydrolases, oxidation–reduction enzymes, transcription factors and so on. In addition, there were 21 putative PHI (Pathogen–Host Interaction) genes out of those 93 up-regulated genes. In this report, we analyzed virulence-associated genes in *I. mors-panacis* G3B which may be related to detoxifying saponins to infect *P. notoginseng* successfully. They provided an excellent starting point for in-depth study on pathogenicity of *I. mors-panacis* G3B and developed appropriate root rot disease management strategies in the future.

## Introduction

*Panax notoginseng* (Burkill) F.H. Chen (*P. notoginseng*) is a kind of traditional Chinese medicinal herb mainly used for treatment of a variety of diseases, such as ischemic cardiovascular diseases and so on. *P. notoginseng* which belongs to the *Panax* genus and Araliaceae has been cultivated for more than 400 years in china since the time of Shizhen Li who compiled “Compendium of Materia Medica”. Currently, it was mainly cultivated artificially in the mountain area with altitude between 1200 and 2000 m of Wenshan prefecture, Yunnan province, China (Guo et al. 2010). Although there had been reports on wild species of *P. notoginseng*, no successful case had been described and only some closely related wild species or variety had been found (Yan et al. 2006). *P. notoginseng* is a perennial medicinal plant that grows in the shade and needs 4–6 years to produce mature roots and accumulate of bioactive compounds such as saponins. The humid production environment and prolonged period of growth expose the roots to potential infection by different pathogens, which severely restrict the production of *P. notoginseng*, especially in land-limited mountain area.

*P. notoginseng* is vulnerable to be attacked by soil microbes including fungi, bacteria and nematodes because of its long-term cultivation and shady environment. Fungi dominate with increasing years of planting, more than 70 genera were found in the rhizosphere soil of *P. notoginseng* cultivated in Wenshan prefecture, and 20 species have been identified (Wang et al. 2003). Pathogenic fungi cause serious diseases, such as black spot, circular spot, gray mold, root rot and so on. Among them, root rot is the most common and severe disease, resulting in dramatic crop losses up to 10–20%, or more than 70% (Wu et al. 2015; Sun et al. 2004). Root rot typically appears at the tip of the taproot destroying the fibrous roots and attacks toward the crown, the core of the root eventually disintegrates and remains hollow, which is also known as disappearing root rot (Rahman and Punja 2005). Thus, identification of the dominant pathogens of the root rot and clarification their pathogenesis are prerequisites for effective control to maintain the sustainable cultivation of *P. notoginseng*.

Root rot is an ubiquitous disease worldwide, occurring in multiple plants with varied pathogens. And *Cylindrocarpon destructans* is a kind of soilborne pathogenic fungi which can cause severe root rot in many hosts including *P. ginseng*. They can be divided into weak and aggressive isolates. However, aggressive isolates cause severe root rot disease especially to *P. gingseng* with limited pathogenicity in other hosts, and were therefore named *C. destructans* f. sp. *Panacis* (Seifert et al. 2003). Then, *C. destructans* was reclassified as *Ilyonectria radicicola* (Chaverri et al. 2011). According to multigene molecular analysis especially histone H3 (HIS H3), *I. radicicola* isolates appeared a polyphyletic relationship and each group of isolates is considered to be a different species (Lombard et al. 2013; Cabral et al. 2012). And *C. destructans* f. sp. *panacis* was genetically distinct from the other isolates and clustered in a distinct group named as *Ilyonectria mors-panacis* (Seifert et al. 2003). On these bases, Mi used culture-dependent and molecular methods to investigate the fungal communities and identify the dominant pathogen of G3B inducing root rot of *P. notoginseng* based on in vitro and in vivo pathogenicity. G3B is phylogenetically and phenotypically similar to *I. mors-panacis*, so named for *Ilyonectria mors-panacis* G3B (Mi et al. 2017). And Zhu reported available genome sequence of *I. mors-panacis* G3B and its annotation, the annotation analysis predicted 120 virulence genes defined by the Database of Virulence Factors in Fungal Pathogens including 28 cell wall degrading enzymes, 18 antioxidant systems, 7 effectors and so on. This further proved that *I. mors-panacis* G3B is the dominant agreesive pathogen causing root rot of *P. notoginseng* (Zhu et al. 2019). Therefore, understanding the genetic properties and pathogenicity of *I. mors-panacis* will provide theortical supports for inhibiting root rot to maintain sustainable cultivation of *P. notoginseng*.

*Ilyonectria mors-panacis* has complicated pathogenicity processes, they mainly include the following steps: when spore or mycelium adheres to the root surface, *I. mors-panacis* rapidly produces high quantities of hydrolytic enzymes, such as cellulase and pectina* mors-panacis*, allowing rapid invasion of the epidermal layer and fast extension of the inoculum to the cortical and inner tissues; And the ginseng plant starts to secret ginsenosides or phenolic compounds to resist the invasion by pathogens. In response to them,* I. mors-panacis* further produces enzymes that can degrade ginsenosides or phenolic compounds, such as glycosidases and polyphenoloxidases. At the same time, *I. mors-panacis* sequesters iron from the ginseng plant to support its growth using siderophores; once the cell wall components of ginseng plant break down, its defense response declines and *I. mors-panacis* propagates quickly and then root rotting symptoms are established (Morrissey et al. 2000; Rahman and Punja 2005; Ivanov and Bernards 2012; Farh et al. 2018). During the pathogenicity processes, ginsenosides secreted by ginseng plant have been shown to possess chemical defenses against fungi and therefore act as phytoanticipins (Nicol et al. 2002). However, the antifungal action of ginsenosides is not effective against all potential ginseng pathogens. Oppositely, they have been shown to stimulate the in vitro growth of some pathogens inducing root rot of ginseng plants and this may result from the ability of these pathogens to metabolize ginsenosides via extracellular glycosidases (Andreea Neculai et al. 2009; LF and MA 2006). In addition, relevant study has shown that the highly aggressive species of pathogens inducing root rot disease produces much more hydrolytic enzyme, the oxidative enzyme and polyphenol oxidase than weakly aggressive, which destroy the plant defensive barriers (Rahman and Punja 2005). However, the detail involvements of hydrolytic enzymes in detoxifying ginsenosides and more related pathogenesis have yet been studied.

Therefore, the goal of the present study is to analyze the genes encoding the saponins degrading enzymes from *I. mors-panacis* G3B. To achieve this objective, we built the transcriptome sequencing platform for high-throughput prediction of all associated genes. This study not only revealed candidate genes for further functional research on pathogenicity of *I. mors-pannacis* but also provided theoretical supports for inhibiting root rot and alleviating replant failure of ginseng plants.

## Materials and methods

### Fungal strains and growth conditions

*Ilyonectria mors-panacis* G3B used in the study was first isolated from the rhizosphere soil of diseased *P. notoginseng* cultivated in Wenshan, Yunnan Province, China (Mi et al. 2017). The strain was cultured onto a PDA (Potato Dextrose-Agar) plate at 22 ℃ for 18 days.

### Assessment of the tolerance of Ilyonectria mors-panacis G3B to saponins

The susceptibility of *I. mors-panacis* G3B to saponins was evaluated by estimating the growth diameters of colonies which were inoculated in PDA agars and those supplemented with Sanqi total Saponins (SAPs) (Solarbio). 5 µL conidial suspension (1 × 10^7^ conidia mL^−1^) was, respectively, applied to the center of PDA agar plates (90 mm in diameter) and those containing SAPs at the concentration of 500 ppm. Inoculated plates were incubated at 22 ℃ and colonies diameters were measured daily from 3 days post inoculation until the colonies ceased growing. Three samples per treatment were used as replicates and the experiment was conducted three times.

### Assessment of the capacities of Ilyonectria mors-panacis G3B to degrade saponins

Conidial suspensions of *I. mors-panacis* G3B were inoculated in the PDA liquid medium supplemented with proper amount of saponins. And non-inoculations of *I. mors-panacis* G3B in the PDA liquid medium containing equivalent saponins were negative controls. According to the standard curves (concentration-peak area) of Rg1 and Rb1 analyzed by HPLC, we assayed the concentrations of Rg1 and Rb1 after 0 day, 3 days and 6–12 days inoculation in the PDA culture solution, respectively.

### *I. mors-panacis* G3B incubation with saponins and RNA extraction

The conidia of *I. mors-panacis* G3B were inoculated in sabouraud dextrose broth at a final concentration of 1 × 10^6^ conidia mL^−1^ and incubated for 36 h at 22℃ with 220 rpm. The mycelium was collected by filtration, and washed three times with sterilized water. Subsequently, 0.5 g mycelia were inoculated into 50 mL basal salt solution (M100 medium with glucose excluded) and equivalent basal salt solution added with saponins as sole carbon and nitrogen sources, respectively, then incubated at 22℃ with 220 rpm for 4H and 12H. The mycelia of *I. mors-panacis* G3B at each time point were collected and rinsed with distilled water and then immediately frozen in liquid nitrogen until RNA extraction. The mycelium harvested from *I. mors-panacis* G3B incubated with saponins-free basal salt solution served as controls (4H and 12H). The total RNA was extracted using TRIzol Reagent in accordance with manufacturer’s protocols and then treated with RNase-free DNase to eliminate genomic DNA contamination. The total RNA was quantified on the Thermo Scientific NanoDrop 2000 spectrophotometer and the Agilent 2100 Bioanalyzer.

### cDNA library construction and sequencing

The mRNA was purified and isolated by treating total RNA with Magnetic Oligo (dT) beads. Then, the purified mRNA was sheared to approximately 200 bp fragments prior to cDNA synthesis. Short fragments were purified and ligated to sequencing adapters. Fragments with suitable sizes on the basis of agarose gel electrophoresis were selected as templates for PCR amplification to isolate and purify the cDNA fragments for sequencing. Construction of libraries (Illumina Truseq™ RNA sample prep Kit) and sequencing with the Illumina HiSeq 2000 platform were performed by Frasergen (Shanghai, China). The quality of raw RNA-Seq reads was filtered using the following criteria: (1) reads including adapter sequencing or empty adapter were filtered; (2) reads for which Ns comprised more than 10% of the total length were discarded; (3) reads with low-quality bases (< Q20) were filtered.

### RNA-Seq reads mapping and annotation

Hisat2 v 2.1.0 was used to map the RNA-Seq reads with the reference genome for subsequent analysis. The mapped reads were subjected to de novo transcriptome assembly using trinity assembly software to obtain high-quality transcript sequence. Then, the assembled sequences were used for a homology search against the NR, String, Swissport and KEGG database by NCBI-Blastx Version 2.2.25 with an E-value of 10^–5^.

### Gene expression analysis and DEGs validation

Reads that aligned uniquely to the reference sequence were used for gene expression quantification that were measured and normalized as the fragments per kilobase of exon per million fragments mapped (FPKM), which is similar to reads per kilobase of exon per million mapped reads (Marioni et al. 2008). Differential expression analysis was performed with edgeR v 3.24 software using the test of fold change (|log_2_FPKM|> 1) and false discovery rate (fdr < 0.05) to estimate the level of differential gene expression by each sample under different induction conditions (Benjamini et al. 2001).

### Analysis virulence-associated genes during saponins metabolism

To identify potential pathogenicity and virulence genes, whole genome blast searches were conducted against protein sequences in the Pathogen–Host Interaction database (PHI database) (version 3.2, http://www.phi-base.org/) (*E* < 1 × 10^–5^). Further, we found the partial virulence-associated genes identified from the PHI database among differential expression genes at 4H and 12H during saponins metabolism.

## Results

### The tolerance of Ilyonectria mors-panacis G3B to saponins

**Fig. 1:**
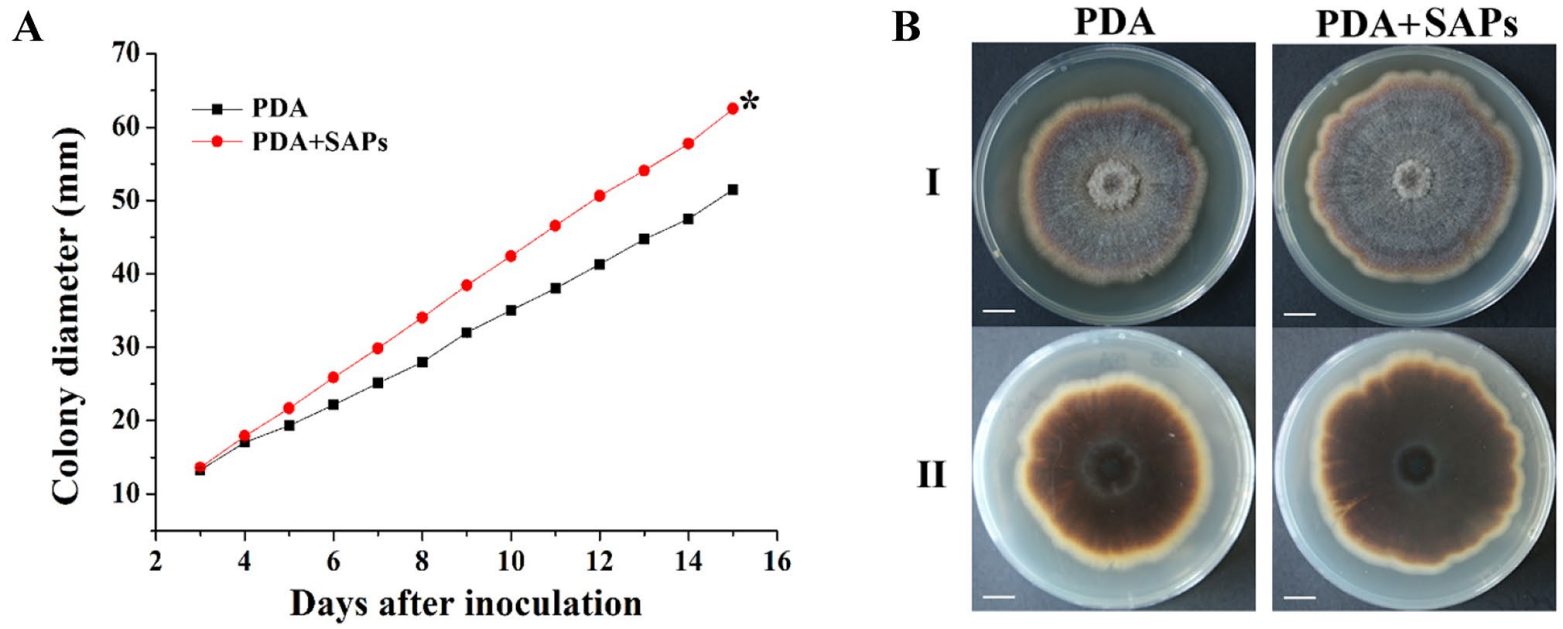
The tolerance of *I. mors-panacis* G3B to Sanqi total Saponins (SAPs). **(A)** Growth curves of *I. mors-panacis* G3B on PDA plates without SAPs and PDA plates supplemented with SAPs (500 ppm). The star indicates that the growth of *I. mors-panacis* G3B on the PDA plates supplemented with SAPs was significantly faster than that on the PDA plates without SAPs (*P* < 0.05). **(B)** Morphology of colonies of *I. mors-panacis* G3B. Colony pictures were taken at 15 days post inoculation by applying 5 μL of a conidial suspension (1 × 10^7^ conidia mL^−1^) inoculated on the PDA without SAPs and PDA supplemented with SAPs (500 ppm). Scale bars represent 10 mm

*P. notoginseng* can secret secondary metabolites such as saponins that used for anti-microbes. Therefore, we assayed the sensitivity to saponins of *I. mors-panacis* G3B as dominant pathogen inducing root rot of *P. notoginseng*. Under saponins stress [PDA supplemented with 500 ppm Sanqi total Saponins (SAPs)], *I. mors-panacis* G3B grew significantly faster and produced bigger isolated colonies than control (Inoculation on PDA without SAPs) (*P* < 0.05) (Fig. 1). They suggested that *I. mors-panacis* G3B could degrade saponins and use them as carbon sources, and this might also be the main factor for *I. mors-panacis* G3B to infect the *P. notoginseng* successfully.

### The capacity of Ilyonectria mors-panacis G3B to detoxify saponins

**Fig. 2:**
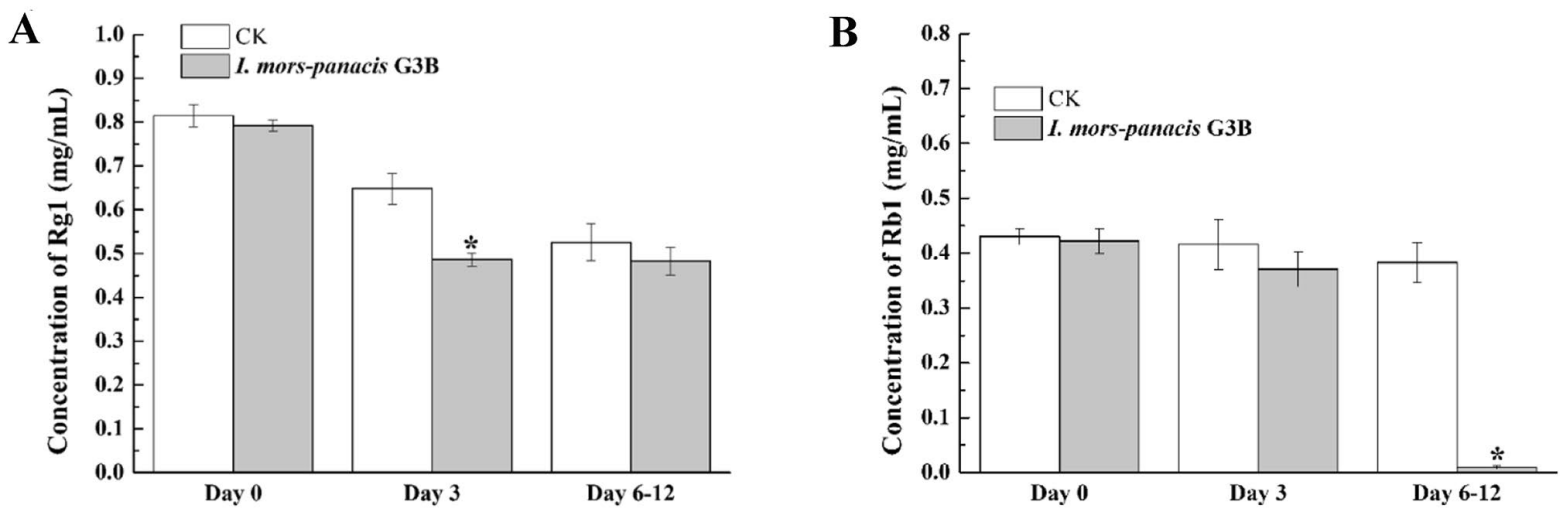
The concentration of Rg1 (A) and Rb1 (B) in the culture solution at different time post inoculation with *I. mors-panacis* G3B or without inoculation (CK). The stars indicate that the concentration of Rg1 and Rb1 in the culture solution inoculation with *I. mors-panacis* G3B was significantly decreased compared with CK (*P* < 0.05). The experiments were repeated three times with three replicates per repeat

To verify the degradation effect on saponins of *I. mors-panacis* G3B directly, we assayed its abilities to degrade the Rg1 and Rb1. Compared with CK, the concentration of Rg1 and Rb1 in the culture solution inoculation with *I. mors-panacis* G3B was significantly decreased at 3 days and 6–12 days post inoculation, respectively (Fig. 2). They indicated that *I. mors-panacis* G3B could degrade different kinds of saponins, such as Rg1 and Rb1, and exhibiting different degradation abilities.

### Overview of RNA-Seq analysis of *I. mors-panacis* G3B

**Fig. 3:**
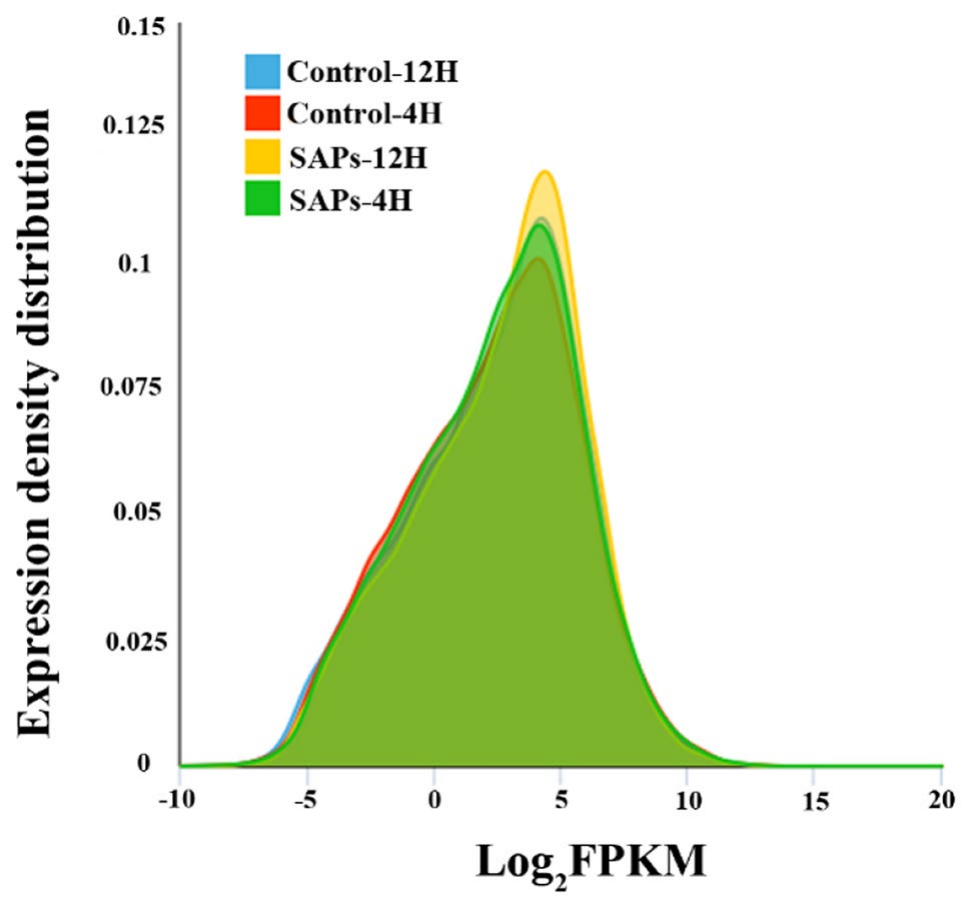
The expression density distribution of *I. mors-panacis* G3B with or without saponins (SAPs) treatments for 4H and 12H

In general, saponins exhibit antifungal activities against soil-borne fungi. However, the pathogenic fungi can produce saponins detoxifying enzymes to decrease the fungi toxicity (Watanabe et al. 2004; Yousef and Bernards, 2006). To find the genes which may be related to detoxifying saponins, the transcriptomes of *I. mors-panacis* G3B which were treated with saponins for 4H and 12H were profiled by Illumina HiSeq 2000 RNA-Seq (free-saponins treatment as negative control), two biological replicates and 8 datasets were established. Approximately 30 million 200 bp paired-end reads were generated, the majority of the reads (~ 94%) were mapped to the *I. mors-panacis* G3B draft reference genome sequence which had been available from the GenBank under the accession number PPHJ00000000.1 (BioProject: PRJNA431033) and more than 70% unique mapped reads appeared, indicating the high abundance and excellent quality of the sequencing data (Table 1). In this study, one gene was considered to be expressed when its fragments per kilobase per million fragments (FPKM) were greater than or equal to 1 and genes with FPKM between 8 and 32 represented a majority (Fig. 3).

**Table 1:** Summary of Illumina sequencing and transcriptome assemblies for RNA-Seq libraries

Samples	Reads	Length	Q20 (%)	Q30 (%)	GC content (%)	Mapping reads (ratio %)	Unique reads (ratio %)
Control-4H	29,058,748	200	98.4	95.4	55	27,463,859 (94.5)	22,698,931 (78.1)
Control-12H	30,631,115	200	98.3	95.3	55.5	28,959,571 (94.5)	26,064,239 (85.1)
SAP-4H	31,643,822	200	98.4	95.5	54.2	29,920,854 (94.6)	23,132,888 (73.1)
SAP-12H	30,085,411	200	98.4	95.6	54.8	28,530,258 (94.8)	23,817,812 (79.2)

### DEGs identification

**Fig. 4:**
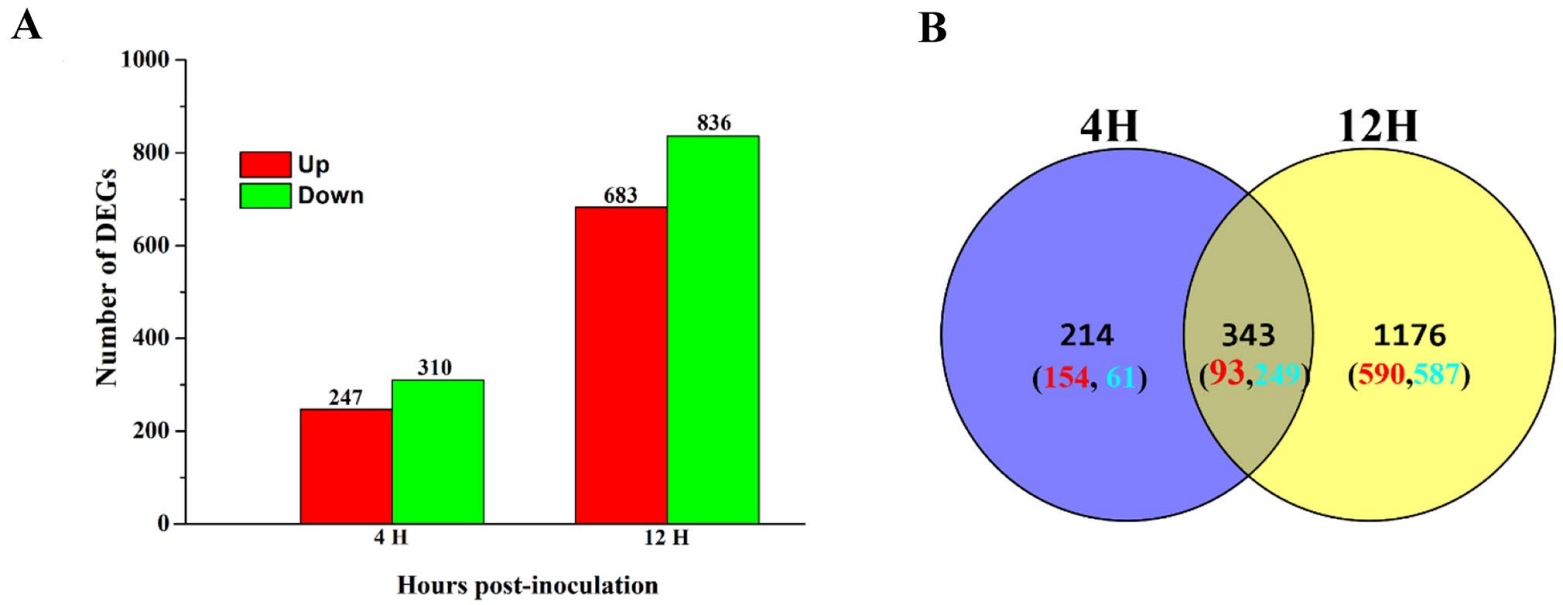
Transcriptomic analysis of *I. mors-panacis* G3B during saponins catabolic process. **(A)** DEGs of mycelium incubated with saponins compared with no saponins treatments for 4H and 12H, respectively. **(B)** Venn diagram showing the distribution of shared DEGs of mycelium incubated for different time courses (4H and 12H). Shown in the parentheses are the number of upregulated genes (red) and downregulated genes (green) in *I. mors-panacis* G3B with saponins treatments for 4H and 12H compared with control (without saponins treatments)

To identify the putative signal transduction and metabolic pathways involved in detoxifying saponins in *I. mors-panacis* G3B, we used RNA-Seq to compare the set of differentially expressed genes (DEGs) between saponins treatments and free-saponins treatments for 4H and 12H, respectively. In this section, ‘up-regulated genes’ are genes with higher expression levels when saponins treatment, and ‘down-regulated genes’ are those with lower expression levels under the same conditions. When mycelium of *I. mors-panacis* G3B grown for 4H and 12H, the number of DEGs was 557 (247 up-regulated and 310 down-regulated) and 1519 (683 up-regulated and 836 down-regulated), respectively. In addition, 343 genes including 93 up-regulated genes and 249 down-regulated genes presented the same expression pattern in the mycelium of *I. mors-panacis* G3B with saponins treatments compared with free-saponins treatments for 4H and 12H (Fig. 4).

### Core genes encoding saponins detoxifying enzymes

**Fig. 5:**
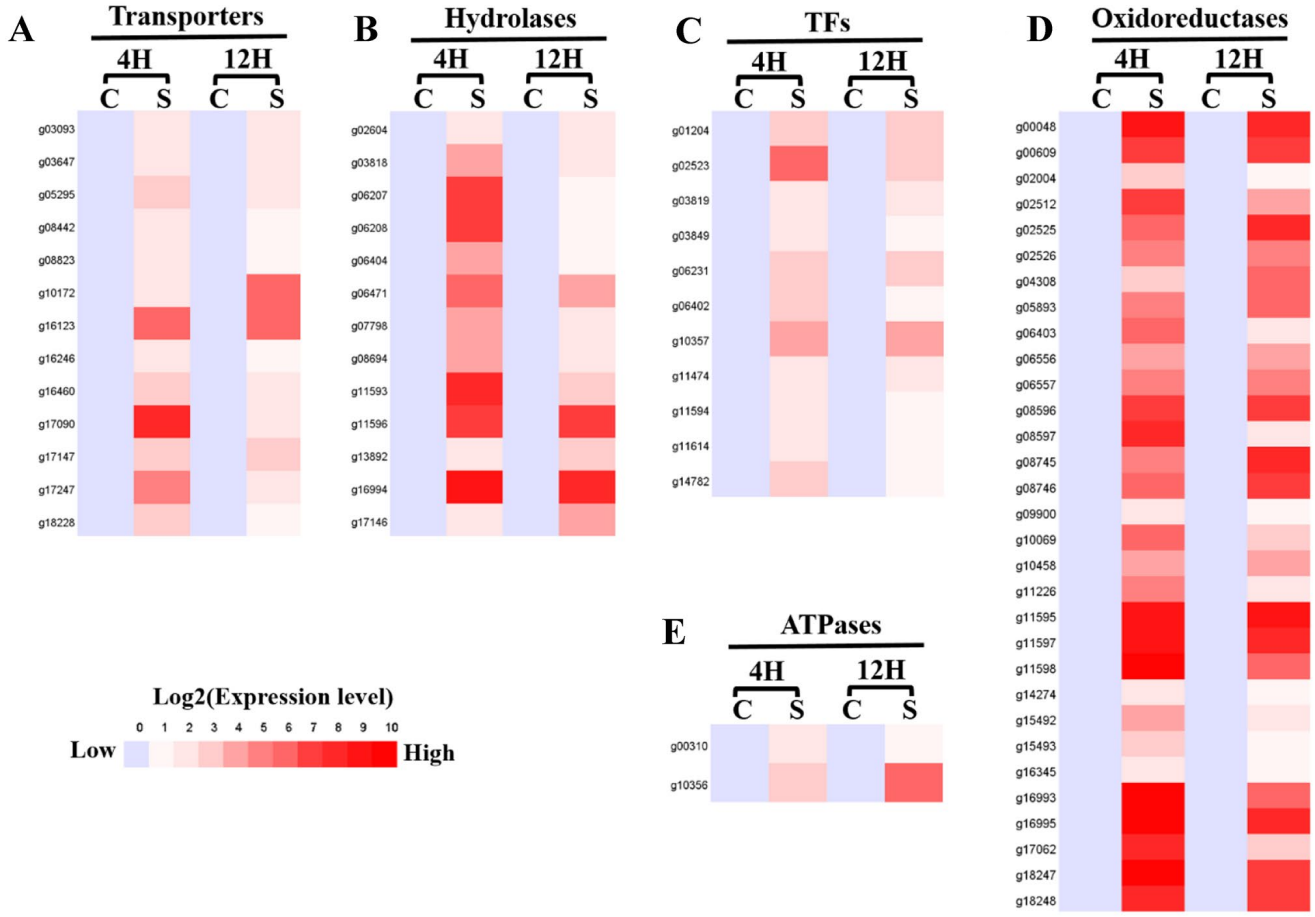
Expression patterns of some transporters (A), hydrolases (B), transcription factors (TFs) (C), oxidoreductases (D) and ATPases (E) in *I. mors-panacis* G3B. C represent controls, *I. mors-panacis* G3B were inoculated in the basal salt solution without saponins for 4H and 12H; S represent samples, *I. mors-panacis* G3B were inoculated in the basal salt solution with saponins for 4H and 12H

Saponins are kinds of glycosides whose aglycones are triterpenes or spiral steranes, they are composed of saponin units and sugars, such as glucose, galactose, rhamnose, arabinose, glucuronic acid, galacturonic acid and so on. And the genes involved in their metabolism are unknown. According to the above RNA-Seq analysis, if a DEG in the *I. mors-panacis* G3B with saponins treatments had a similar expression pattern between 4 and 12H post inoculation, it may be involved in signal transduction and metabolic pathway of saponins. Therefore, we mainly focused on the above 93 up-regulated genes simultaneously present in the *I. mors-panacis* G3B treated with saponins for 4H and 12H, they mainly included several kinds of transporters, glycoside hydrolases, oxidation–reduction enzymes, transcription factors and so on (Fig. 5). We speculated that they are responsible for transporting and metabolizing saponins, making *I. mors-panacis* G3B resist the antimicrobial activity of saponins and infect *P. notoginseng* successfully.

### Virulence-associated genes

To find potential virulence-associated genes, the whole genome blast analysis was conducted against the Pathogen–Host Interaction (PHI) gene database, a collection of experimentally verified pathogenicity, virulence and effector genes from fungi, oomycetes and bacteria (Winnenburg et al. 2007). And we identified 2298 putative PHI genes in *I. mors-panacis* G3B (12.5% of its genes). We processed on the assumption that the proof of pathogenicity or virulence of a gene in one fungus may also suggest a pathogenicity or virulence in other fungi (Baldwin et al. 2006). Therefore, the 2298 putative PHI genes may be involved in pathogenicity in *I. mors-panacis* G3B, and we found 21 genes which were simultaneously highly expressed in *I. mors-panacis* G3B treated with saponins for 4H and 12H among them (Table 2). They may be pathogenicity determinants and involved in detoxifying saponins as antimicrobial compounds.

**Table 2:** Statistic of virulence-associated genes

Gene	Log_2_ (Fold Change) (4H/12H)	Description	PHI accession number
g03093	2.72/2.58	Facilitated glucose transporter	PHI:538|FRT1|AAU87358|TX:40,559|Botrytis
g05295	3.51/2.96	Hexose transporter protein	PHI:538|FRT1|AAU87358|TX:40,559|Botrytis
g08442	2.55/1.71	Oligopeptide transporter	PHI:1085|Ptr2|AAO31597|TX:13,684|Stagonospora
g10172	2.81/6.87	Cycloheximide resistance protein	PHI:26|CaMDR1|CAA37820|TX:5476|Candida
g16123	6.67/6.61	abc-2 type transporter	PHI:258|GPABC1|CAC40023|TX:5128|Gibberella
g17147	3.57/3.89	Sugar transporter	PHI:538|FRT1|AAU87358|TX:40,559|Botrytis
g17247	5.14/2.13	Polyamine transporter 4	PHI:26|CaMDR1|CAA37820|TX:5476|Candida
g06404	4.12/1.71	Glycosyl hydrolases family 18 protein	PHI:144|CHT42|AAC05829|TX:29,875|Trichoderma
g06471	6.86/4.78	Cutinase 3	PHI:407|PBC1|CAB40372|TX:76,659|Pyrenopeziza
g17146	2.84/4.57	Triacylglycerol lipase	PHI:541|LIP1|AAU87359|TX:332,648|Botrytis
g02004	3.97/1.37	n-alkane-inducible cytochrome p450	PHI:438|BcBOT1
g02512	7.71/4.97	Alcohol dehydrogenase	PHI:881|MGG_04556|EDJ96020|TX:318,829|Magnaporthe
g10458	4.04/4.13	Cytochrome p450	PHI:438|BcBOT1
g11226	5.08/2.71	Aldehyde reductase II	PHI:1047|CTB6|ABK64183|TX:29,003|Cercospora
g14274	2.20/1.96	Restculine oxidase	PHI:716|ZEB1|ABB90284|TX:5518|Fusarium
g00310	2.33/1.76	Leptomycin b resistance protein pmd1	PHI:1018|ABC3|AAZ81480|TX:318,829|Magnaporthe
g10356	3.48/6.24	Atpase	PHI:132|ABC1|AAB86640|TX:318,829|Magnaporthe
g02523	6.05/3.84	Cutinase transcription factor 1 beta	PHI:1021|CTF1|ABR12478|TX:5507|Fusarium
g03819	2.90/2.14	Cutinase transcription factor 1 beta	PHI:1021|CTF1|ABR12478|TX:5507|Fusarium
g06402	3.00/1.77	Fungal specific transcription	PHI:1021|CTF1|ABR12478|TX:5507|Fusarium
g11614	2.57/1.65	Fungal specific transcription factor domain-containing protein	PHI:1021|CTF1|ABR12478|TX:5507|Fusarium

## Discussion

Ginsenosides are antifungal compounds that are thought to be secreted by ginseng plants to defense infection by soilborne fungi (Augustin et al. 2011), their concentrations dramatically increase in adventitious hairy roots of *Panax ginseng* when attacked by microbes (Liu et al. 2004). However, ginsenosides showed a rapid reduction in roots infected with the aggressive *I. mors-panacis* isolates (Farh et al. 2017). Relevant study also showed that when the tomato was artificially inoculated with *Cladosporium fulvum* inducing blight spot, α-tomatine secreted by tomato plants reduced because of β-glucosidase produced by pathogen hydrolyzing it, resulting in accumulation of a less fungi-toxic compound (Okmen et al. 2013). In the current study, we found the growth of *I. mors-panacis* G3B was significantly increased in media supplemented with saponins, indicating that *I. mors-panacis* G3B could produce associated saponin-hydrolyzing enzymes to metabolize saponins. Therefore, *I. mors-panacis* may use a similar mechanism to detoxify ginsenosides, such as Rg1 and Rb1 and so on, and resist antifungal activity to infect the ginseng plants successfully.

Recent improvements in next-generation sequencing technology and bioinformatics now allow the de novo assembly of high-quality eukaryotic genome (Nowrousian et al. 2010; Li et al. 2010). Previously, we used such an approach to provide the first draft sequences of *I. mors-panacis* G3B inducing root rot of *P. notoginseng*, and thus serve as an excellent starting point for gaining a broad perspective of issues in *P. notoginseng* pathology (Zhu et al. 2019). In this study, we used high-throughput RNA-Seq to characterize the transcriptome profile of *I. mors-panacis* G3B with saponins treatments for different time periods. The induced responses of *I. mors-panacis* G3B to saponins treatments were characterized to reveal genes involved in the saponins detoxification. Saponins, based on a dammarane carbon skeleton with four trans-oriented rings and side chains that consist various sugar moieties (mono- and disaccharides of glucose, rhamnose, xylose and arabinose) attached through the C-20 and either the C-3 or C-6 positions (Attele et al. 1999). By comprehensive analysis, a total of 93 unigenes were all up-regulated in mycelium harvested from *I. mors-panacis* G3B treated with saponins for 4H and 12H. The above-related gene encoding proteins mainly belong to transporters, glycoside hydrolases, oxidation–reduction enzymes, transcription factors and therefore they may be involved in putative signal transduction and metabolic pathways to detoxify saponins.

Using the experimentally verified Pathogen–Host Interaction (PHI) gene reference database (Winnenburg et al. 2007), we found that 12.5% of the genes (2298) in the *I. mors-panacis* G3B genome have significant similarities with genes involved in pathogenicity in other fungi, such as plant pathogens *F. graminearum* and *M.oryzae* and even animal pathogens *C. albicans*, they may be candidate genes controlling pathogenicity. Further, we identified 21 genes which were simultaneously highly expressed in *I. mors-panacis* G3B treated with saponins for 4H and 12H among the 2298 genes, they may be involved in saponins metabolism for resisting antifungal activity to control pathogenicity.

In conclusion, our study explored the tolerance of *I. mors-panancis* G3B to saponins, which may intervene in the plant defense mechanism against pathogens infecting ginseng plants. The pathogenicity of *I. mors-panacis* G3B may mainly depended on detoxification saponins and we analyzed associated virulence genes which may be involved in saponins metabolism. They provide an excellent starting point for in-depth study of biological function of related genes in the further work and gain advanced insights into the pathogenicity of *I. mors-panacis* causing root rot in ginseng plants.
